# Development and Validation of the Brief Folate-Specific Food Frequency Questionnaire for Young Women’s Diet Assessment

**DOI:** 10.3390/ijerph14121574

**Published:** 2017-12-14

**Authors:** Dominika Głąbska, Aneta Książek, Dominika Guzek

**Affiliations:** 1 Department of Dietetics, Faculty of Human Nutrition and Consumer Sciences, Warsaw University of Life Sciences (WULS-SGGW), 159c Nowoursynowska Street, 02-776 Warsaw, Poland; student_WULS@wp.pl; 2 Department of Organization and Consumption Economics, Faculty of Human Nutrition and Consumer Sciences, Warsaw University of Life Sciences (WULS-SGGW), 159c Nowoursynowska Street, 02-776 Warsaw, Poland; dominika_guzek@sggw.pl

**Keywords:** folate, food frequency questionnaire, validation study, validity, reproducibility, young women

## Abstract

The tools enabling brief assessment of folate intake may be of great value for public health purposes. The aim of the presented study was to design a brief folate-specific food frequency questionnaire for Central and Eastern European population of women, as well as to assess the validity and reproducibility of the designed Folate-Intake Calculation-Food Frequency Questionnaire (Fol-IC-FFQ) on a group of Polish women aged 20–30 years. Participants collected 3-day dietary records and completed the Fol-IC-FFQ twice (FFQ1: directly after the dietary record; and FFQ2: six weeks later). The analysis included an assessment of validity (comparison of the results of FFQ1 and 3-day dietary record) and of reproducibility (comparison of the results of FFQ1 and FFQ2). In assessment of validity, a Bland-Altman index of 5.3% was observed. In assessment of reproducibility, a Bland-Altman index of 2.7% was observed, the share of individuals classified into the same intake adequacy category was over 85%, the share of individuals classified into the same tertile was almost 75%, the weighted κ statistic indicated substantial agreement (0.67) and correlation was significant (*p* = 0.0000; *R* = 0.7995). Assessment of the Fol-IC-FFQ revealed a satisfactory level of validity and very good level of reproducibility in the population of young Polish women. The Fol-IC-FFQ may be considered a valid tool for the assessment of folate intake in young Polish women and a promising tool for the assessment of folate intake in young women in Central and Eastern Europe.

## 1. Introduction

Folate is indicated by the World Health Organization (WHO) [1] as a nutrient especially important for women of reproductive age, in particular during pregnancy. A deficiency of this nutrient is associated with neural tube defects in progeny, and also with anemia in young women [2]. Considering both neural tube defects and anemia prevention in pregnant women, it is recommended to implement folic acid supplementation as early as possible, while supplementation before conception is indicated as the best option [2].

In terms of WHO’s global targets to be achieved by the year 2025 [3], the role of folate is also undeniable. Regarding the global target of the reduction of anemia in women of reproductive age and of the reduction of low birth weight, there is direct evidence of the effect of daily iron and folic acid supplementation during pregnancy, from the review and meta-analysis of Peña-Rosas et al. [4]. In terms of the global target of the reduction of the number of children who suffer from stunted growth, as well as the reduction of childhood wasting, there is no direct evidence for the link between supplementation during pregnancy and the achievement of these goals, but the pooling analysis of longitudinal birth cohorts conducted by Christian et al. [5] enabled conclusions to be drawn about existing associations [6].

However, it must be indicated that in Western countries, both before and during pregnancy, a number of women do not take adequate folic acid supplements, and this is especially alarming, if in a given country there is no mandatory folic acid fortification [7]. Among countries without such fortification requirements are the Netherlands and Norway, where the frequency of non-adherence to recommended supplementation in the preconception period is, respectively, 45–63% [8,9] and 70% for women of European ethnicity [10].

Among countries with obligatory folic acid fortification are the United States of America and Canada [11], which is noted as a positive example of a decreased frequency of neural tube defects in progeny [7]. However, in Canada 40% of women still do not achieve the recommended folate blood levels [12]. Taking this deficiency into account, in Canada not only is fortification mandatory and supplementation recommended, guidelines for pregnant women also include dietary counseling associated with the food sources of folate [13].

Such nutritional counseling may be of great value for young women, not only to indicate necessary dietary modifications, but also to make women aware of the need for supplementation, due to their insufficient dietary intake. Especially in non-pregnant women of reproductive age, such education could be valuable to ensure necessary understanding before conception [14]. Considering the fact that the number of unplanned pregnancies is still high [15] and such pregnancies are the main reason for a lack of folic acid supplementation [16], properly planned education should be conducted not only for pregnant women but for all women of reproductive age.

In assessments of folate [17] and vitamin B intake [18] in countries with no folate fortification [19], food frequency questionnaires are suggested as a good option. However, the majority of folate food frequency questionnaires validated so far consist of a large number of questions, and as a result, are time consuming [20]. Simultaneously, brief questionnaires are indicated as especially valuable, as they enable immediate feedback to respondents, indicating their required dietary goals, as well as may be positively validated [21].

The aim of the presented study was to design a brief folate-specific food frequency questionnaire, as well as to assess the validity and reproducibility of the designed Folate-Intake Calculation-Food Frequency Questionnaire (Fol-IC-FFQ) on a group of Polish women aged 20–30 years.

## 2. Materials and Methods

The study was conducted according to the guidelines laid down in the Declaration of Helsinki, and all procedures involving human subjects were approved by the Bioethical Commission of the National Food and Nutrition Institute in Warsaw (No. 0701/2015).

### 2.1. Designing the Folate-Specific Food Frequency Questionnaire Fol-IC-FFQ (Folate-Intake Calculation-Food Frequency Questionnaire)

The designed folate-specific food frequency questionnaire (Fol-IC-FFQ) included only food products that were sources of folate, because the questionnaire was planned as a brief tool to assess only folate intake. All food products characterized by a folate content no lower than 0.1 µg per 100 g were chosen based on Polish food composition tables [22], containing 932 food products and dishes, while both typical and specific local food products are included. Folate was first included in the tables in the year 2000 and there are no missing data for folate. The Polish food composition tables [22] are elaborated by the National Food and Nutrition Institute in Warsaw, which is a member of EuroFIR [23].

All food products meeting the assumed criteria were included in the designed questionnaire, where they were grouped into food product groups characterized by similar folate contents, as is the case in other previously published validation studies [24,25,26]. Afterwards, the food products were clustered into 25 food product groups, while vegetables were clustered into 3 separate groups. This was connected with the fact that in Poland, vegetables and cereals have been listed as the main folate sources [27], and that based on recent data, an even higher contribution from vegetables (35%) than cereals (32%) has been demonstrated [28]. Similarly, in the European Prospective Investigation into Cancer and Nutrition (EPIC) study [29] conducted in 10 European countries, for 8 of them, in groups of women, vegetables were the main folate source, while cereals were ranked second. Simultaneously, for vegetables, a varying folate content is stated. Consequently, 3 groups, characterized by high, medium and low folate content, were created.

The most popular serving sizes were based on the Polish food model booklet [30], as well as on our own previous studies of food frequency questionnaires [24,25,26] and then verified by a dietitian during the pilot research. In the Fol-IC-FFQ these sizes were indicated both in grams and as the described serving sizes.

The designed Fol-IC-FFQ is presented in Table 1 and does not include the folate content in the servings. This is so as not to interfere with the provision of answers. However, for each serving size and for a group of food products specified in the questionnaire, the average folate content was calculated based on the Polish food composition tables [22], as presented in Table 2.

The Fol-IC-FFQ included questions about the exact typical number of servings of products from the indicated food product groups which had been consumed during a typical week or month (open-ended question). The frequency per week or month was chosen based on typical dietary habits in Poland, depending on the type of food product. The frequency per day was not applied to simplify the questionnaire and to avoid excessive overestimation. Considering the fact that vegetables are among the most important sources of folate [22] and that the intake of vegetables depends on the season of the year, information on intake throughout the previous year was required. In the Fol-IC-FFQ, it was also specified that the number of servings should include the servings of products consumed and added to consumed dishes, and it was possible to indicate not only whole integers but also decimal fractions. A similar approach had been adopted in our own previous studies conducted for vitamin D [24], iron [25] and iodine [26].

The procedure for daily folate intake calculation included dividing the number of servings into 7 days a week or 30 days a month (depending on the type of food product) to obtain the daily number of servings. Afterwards, the daily folate intake from specific food product groups was to be estimated using the following formula: folate intake (µg) = daily number of servings × typical folate content in one serving (Table 2). The total daily folate intake was to be obtained as the sum of the values of folate intake from all the groups of products.

### 2.2. Validation of the Fol-IC-FFQ

**Figure 1:**
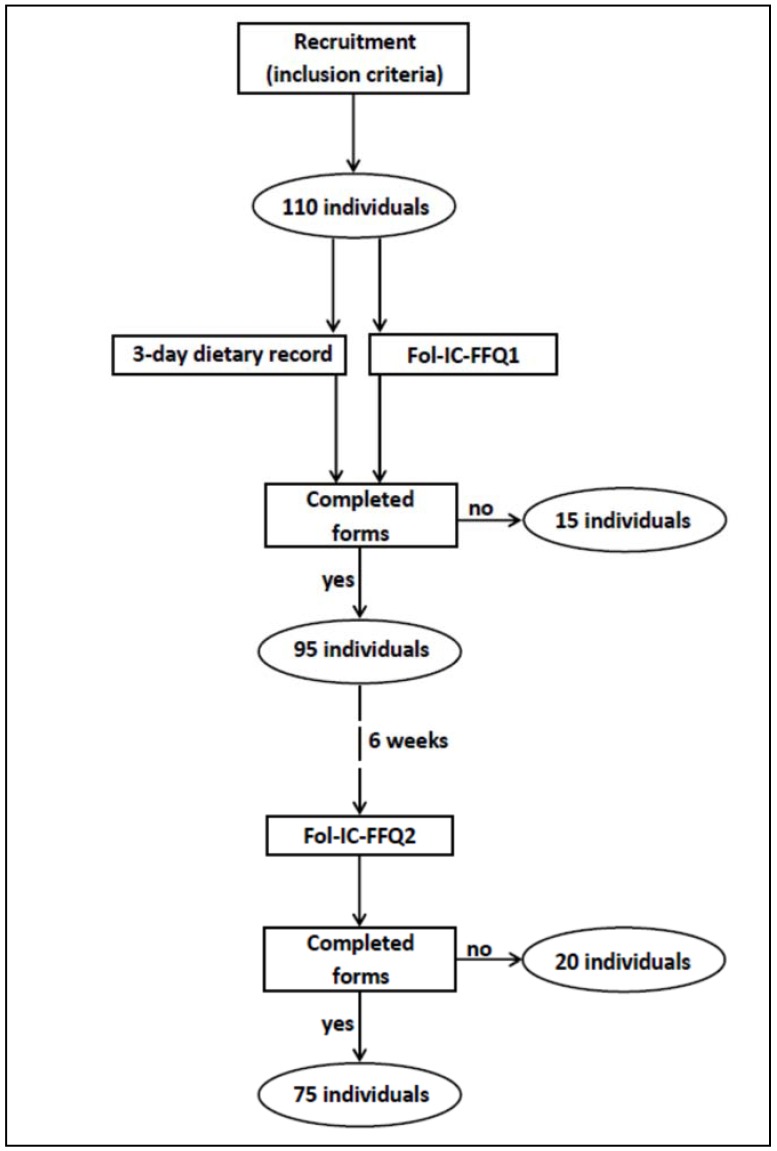
The design of the study.

The Fol-IC-FFQ was validated with a group of young women, and convenience sampling was applied. The inclusion criteria were as follows: women, aged 20–30 years, not undergoing weight loss or on any special diet, not pregnant and not during lactation, without any chronic diseases, and living in Warsaw. There were 110 individuals meeting the inclusion criteria who volunteered to participate in the study. Finally, validation of the assessed Fol-IC-FFQ was conducted with a group of 75 young women, as 35 of the individuals who had initially volunteered did not complete all of the required elements (Figure 1). The obtained sample size was in accordance with recommendations on sample size for validation studies of food frequency questionnaires, as the guidelines state that at least 50 to 100 subjects for each demographic group is recommended [31].

The study of validation was conducted in autumn over a period of 4 months: from September 2016 to December 2016. During this period, the participants were asked to conduct 3-day dietary records and to complete the Fol-IC-FFQ twice (FFQ1: directly after conducting the 3-day dietary record; and FFQ2: 6 weeks after FFQ1). In the analyzed group of young women, about 3–5 min was needed to complete the paper Fol-IC-FFQ form, with 25 questions about product groups and no additional questions included.

Validation of the Fol-IC-FFQ was conducted according to the same methodology as validations published previously [24,25,26]. This included an analysis of the validity (external validation comparing results of FFQ1 with results of the 3-day dietary record, where both assessments were conducted by the same researcher) and reproducibility of the method (so-called repeatability; internal validation comparing results obtained twice—FFQ1 and FFQ2, with both assessments conducted by the same researcher), as defined by Willett and Lenart [32].

Because the aim of the study was to validate a questionnaire that enables assessment of dietary folate intake, the reference value was not folate status, but folate intake. It is indicated that when the food frequency questionnaire is applied for folate assessment and biomarkers are used as reference values, observed errors of determination are related to other factors than error of the data obtained using the food frequency questionnaire [33,34], such as the differing bioavailability levels of folate from various sources, or inadequate folate values in the food composition tables [35]. Taking this into account, it was decided to treat the 3-day dietary record as the reference value for folate intake to obtain a reliable assessment of the questionnaire. Both the 3-day dietary record and the Fol-IC-FFQ assessments were based on self-reported data.

For the 3-day dietary record, the basis of the analysis was a record conducted in three typical, randomly chosen and not successive days (2 weekdays and 1 day of the weekend). The dietary record was conducted on the basis of widely accepted and applied rules: using a structured format, with additional questions about the name of the meal, time and location of consumption, meal ingredients and weight of serving (weighted using kitchen scales) or size of serving (estimated using standard household measures) [36]. To provide reliable estimates of food intake, participants were instructed on the principles of keeping their dietary record as well as on the necessity of accurate and scrupulous recording of all food products consumed and beverages drunk, while the serving sizes were verified afterwards by a dietitian using the Polish food model booklet [30]. Folate intake was analyzed using Polish dietary software “Dietetyk 2” and the Polish database for the nutritional value of products (National Food and Nutrition Institute, 2001) [22].

### 2.3. Statistical Analysis

The statistical analysis of validation included:

- Analysis of the Bland-Altman plots in the assessment of validity (Fol-IC-FFQ1 vs. 3-day record) and of reproducibility (Fol-IC-FFQ1 vs. Fol-IC-FFQ2): the results were interpreted using the Bland-Altman index, whereas the limits of agreement value (LOA) was calculated as the sum of the mean absolute differences of folate intake measured by the two methods, and the ± standard deviation of the absolute difference of folate intake recorded for the two methods magnified by 1.96. In the analysis conducted with the Bland-Altman method to assess agreement between the measurements, a Bland-Altman index of a maximum of 5% (95% of individuals observed to be within the LOA) was interpreted, as commonly assumed [37], as positive validation of the method of measurement.
- Assessment of the share of individuals classified into the same tertile and misclassified (classified into opposite tertiles) in the assessment of validity (Fol-IC-FFQ1 vs. 3-day record) and of reproducibility (Fol-IC-FFQ1 vs. Fol-IC-FFQ2).
- Calculation of the weighted κ statistic with linear weighting to indicate the level of agreement between the classifications into tertiles in the assessment of validity (Fol-IC-FFQ1 vs. 3-day record) and of reproducibility (Fol-IC-FFQ1 vs. Fol-IC-FFQ2). According to the criteria of Landis & Koch [38], values <0.20 were treated as slight agreement, 0.21–0.40 as fair, 0.41–0.60 as moderate, 0.61–0.80 as substantial, and 0.81–1.0 as almost perfect agreement.
- Assessment of the share of individuals classified into the same category (both of either adequate or inadequate intake) and of the conflicting intake adequacy category (adequate intake and inadequate intake) in the assessment of validity (Fol-IC-FFQ1 vs. 3-day record) and of reproducibility (Fol-IC-FFQ1 vs. Fol-IC-FFQ2). Adequate intake was defined according to the Polish recommendations for women on the Estimated Average Requirement (EAR) level as 320 µg [39], which was higher than the level of 250 µg indicated by the European Food Safety Authority (EFSA) [40] as the Average Requirement, but simultaneously this was the same as the EAR level recommended by the Institute of Medicine/National Academy of Medicine [41].

As the supplementary methods in the assessment of reproducibility (Fol-IC-FFQ1 vs. Fol-IC-FFQ2) were conducted:

- Calculation of the root mean square errors of prediction (RMSEP) and median absolute percentage errors (MdAPE) of folate intake.
- Analysis of the correlations between results: the normality of distribution of the results was analyzed using the Shapiro-Wilk test and then Spearman’s rank correlation was applied for nonparametric distribution. Due to the nonparametric distribution, while data were presented, they were compared using the U Mann-Whitney test.

The level of significance was accepted as *p* ≤ 0.05. Statistical analysis was carried out using Statistica software version 8.0 (StatSoft Inc., Tulsa, OK, USA) and Bland-Altman Statistica software macro by Matt Coates, version 2009 (StatSoft Inc., Tulsa, OK, USA).

## 3. Results

Folate intake calculated in the analyzed group, using the 3-day dietary record and Fol-IC-FFQ conducted twice (Fol-IC-FFQ1, Fol-IC-FFQ2), is presented in Table 3. While using the 3-day dietary record, as well as Fol-IC-FFQ, the observed folate intake for the majority of the analyzed group was stated to be inadequate (lower than the Estimated Average Requirement level of 320 µg a day).

**Figure 2:**
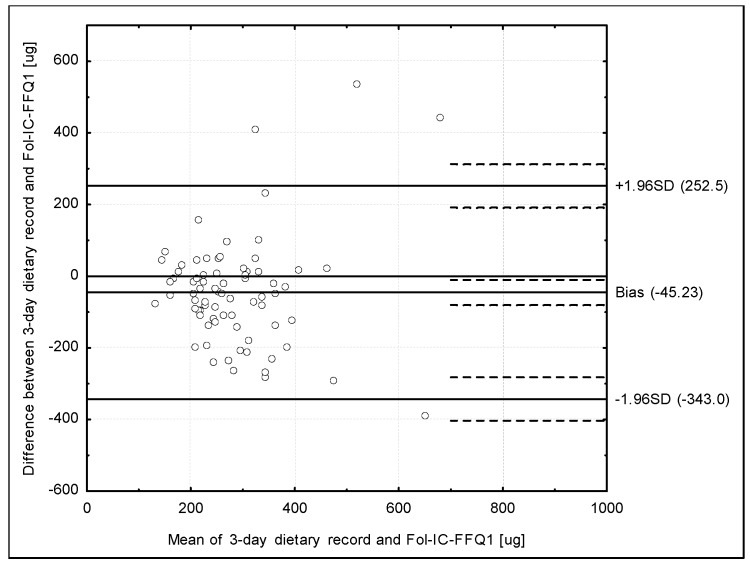
Bland-Altman plot comparing Fol-IC-FFQ1 with 3-day dietary record folate daily intake in the assessment of validity (Bland-Altman index of 5.3%). Fol-IC-FFQ1: food frequency questionnaire filled out directly after conducting 3-day dietary record.

The Bland-Altman plot comparing Fol-IC-FFQ1 with the 3-day dietary record daily folate intake is presented in Figure 2. The mean absolute difference in folate intake was observed to amount to −45.23 µg. After adding ±1.96 standard deviation for the LOA, an interval from −343.0 µg (lower agreement limit) to 252.5 µg (upper agreement limit) was obtained. The number of individuals observed to be within the LOA value was 71 out of 75, which confirmed a Bland-Altman index of 5.3%.

**Figure 3:**
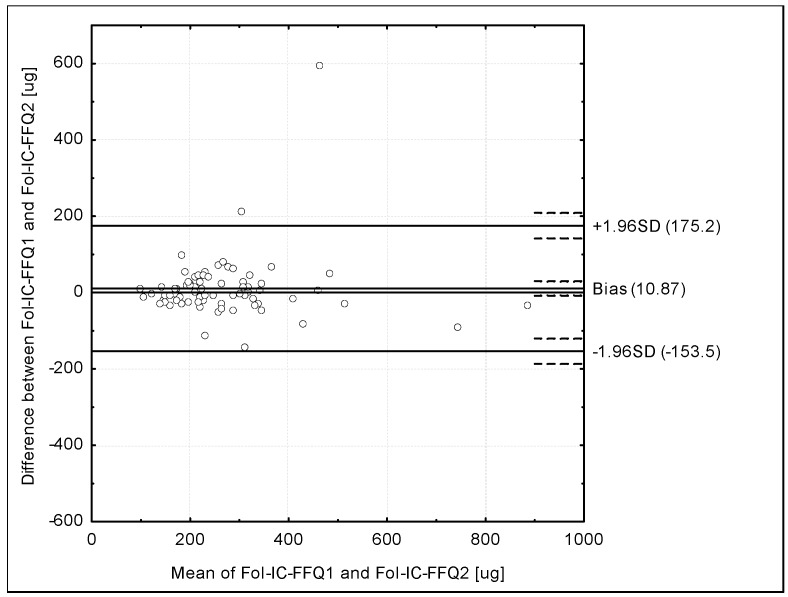
Bland-Altman plot comparing Fol-IC-FFQ1 with Fol-IC-FFQ2 folate daily intake in the assessment of reproducibility (Bland–Altman index of 2.7%). Fol-IC-FFQ1: food frequency questionnaire filled out directly after conducting 3-day dietary record; Fol-IC-FFQ2: food frequency questionnaire filled out six weeks after Fol-IC-FFQ1.

The Bland-Altman plot comparing Fol-IC-FFQ1 with Fol-IC-FFQ2 daily folate intake is presented in Figure 3. The mean absolute difference in folate intake was observed to amount to 10.87. After adding ±1.96 standard deviation for the LOA, an interval from −153.5 µg (lower agreement limit) to 175.2 µg (upper agreement limit) was obtained. The number of individuals observed to be within the LOA value was 73 out of 75, which confirmed a Bland-Altman index of 2.7%.

The share of individuals classified into the same folate intake adequacy category and to the same tertile in the validation of the Fol-IC-FFQ, as well as the weighted κ statistic, are presented in Table 4. The share of individuals classified into the same category in the assessment of validity was over 70%, while in the assessment of reproducibility this result was even higher (over 85%). Simultaneously, in the assessment of reproducibility the share of individuals classified into the same tertile was almost 75% and the weighted κ statistic indicated substantial agreement (0.67).

**Figure 4:**
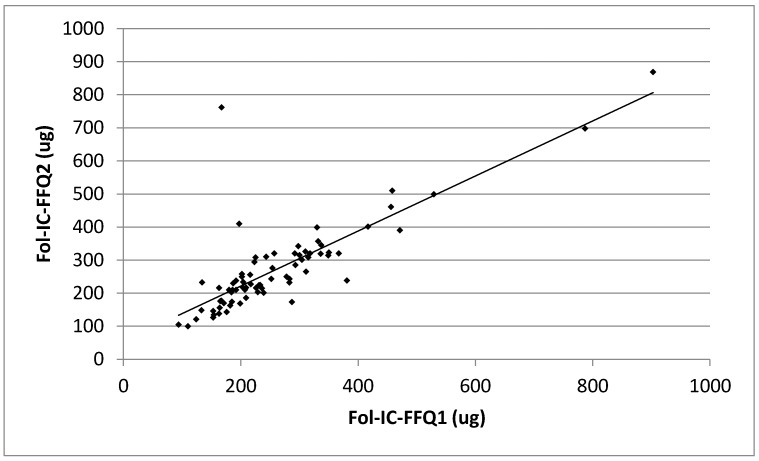
Analysis of the correlation between the Fol-IC-FFQ1 and Fol-IC-FFQ2 daily folate intake (Spearman rank correlation coefficient; *p* = 0.0000, *R* = 0.7995). Fol-IC-FFQ1: food frequency questionnaire filled out directly after conducting 3-day dietary record; Fol-IC-FFQ2: food frequency questionnaire filled out six weeks after Fol-IC-FFQ1.

In the assessment of reproducibility, the RMSEP of folate estimation was 83.99 µg, when the food frequency assessment was conducted twice during a period of six weeks. The MdAPE of folate intake for the comparison between Fol-IC-FFQ1 with Fol-IC-FFQ2 was 10.44%. Simultaneously, the correlation between Fol-IC-FFQ1 with Fol-IC-FFQ2 folate intakes, presented in Figure 4 was significant (*p* = 0.0000; *R* = 0.7995).

## 4. Discussion

Among the factors that influence folate status, as indicated by WHO [1], are diet, physiological status (age, pregnancy or lactation), as well as co-morbidities or socioeconomic status. However, low dietary intake and lack of supplementation are the prime reasons for folate deficiency. Consequently, tools enabling a rapid assessment of intake may be of great value for public health purposes.

Comprehensive questionnaires enabling the assessment of the intake of a few nutrients or many nutrients are commonly designed for B vitamins, including folate. However, the number of food products included in such questionnaires may be very high. Such questionnaires, covering inter alia folate intake assessment, commonly include over 100 items [42,43,44,45,46], or even over 200 items, while it is stated that completing a 235-item questionnaire adapted by Fayet [20] from the 145-item Blue Mountain Eye Study Food Frequency Questionnaire [47] takes approximately 45 min [20].

In order to ensure brief assessment, fewer questions must be included, as is in brief food frequency questionnaires that in general include less than 50 food products [48]. For the 19-question, one-page Block Dietary Folate Equivalents (DFE) Screener, designed to assess folate intake based on the intake of food products contributing to the top 60% of folate intake [49], it is estimated that 6–12 min is enough time to complete the questionnaire [50]. The 19 food product groups of the DFE Screener is similar to the number in conducted own study, as 25 food product groups were included in the Fol-IC-FFQ, but a different approach was applied, as all food products characterized by a folate content no lower than 0.1 µg per 100 g were included and clustered into groups.

The lower number of questions included in brief food frequency questionnaires is also essential to obtain a lower level of overestimation, as was emphasized by van de Rest et al. [45], who stated that a higher number of questions is associated with a higher level of overestimation. As a result, in their food frequency questionnaire applied to assess folate intake, indicated authors included 89 questions, but did not ask about specific vegetables, fruits and beverages intake, but rather asked about total intake of food products from the mentioned groups [51]. The clustering of products from groups characterized by a similar folate content was applied in presented own study for the Fol-IC-FFQ, but for vegetables, three separate groups were identified, based on varying folate contents. It may be stated that the general low number of questions in the Fol-IC-FFQ was the reason for the observed slight underestimation.

In spite of the number of questionnaires designed and validated so far, no similar brief questionnaire for folate intake has been designed for and validated in the countries of Central or Eastern Europe. The majority of brief questionnaires have been validated elsewhere—United States of America [50], Canada [52], Mexico [53,54], Japan [55], Iran [56]. Among European countries, they have been validated for the countries of Northern Europe (Great Britain [57], Norway [35]), Southern Europe (Serbia [17], Croatia [33,34]), as well as Western Europe (The Netherlands [51]), but not for the countries of Central or Eastern Europe.

The lack of a food frequency questionnaire aimed at Central and Eastern European populations may be an important limitation for folate intake assessment in these populations. Folate is indicated as a nutrient especially prone to error in the estimation of its intake, and that is influenced by the fact that there are many plant and animal products which are folate sources and so food product lists applied in such questionnaires must be specific for the population [55]. In various populations, there are some similarities, but also differences in food product choice, and the products commonly consumed in the countries of Western Europe may be different than those in the countries of Central or Eastern Europe. This is especially so for vegetables, which are the main contributor to folate intake, and where the region may be an important influencing factor.

Moreover, while a food frequency questionnaire is designed to assess the intake of only one nutrient (as for brief questionnaires, including Fol-IC-FFQ), it is possible to include the major sources that are most important for a given population [57]. The inclusion of fewer food products in a brief food frequency questionnaire designed to assess the intake of a single nutrient may be better than a higher number, as can be observed in the study by French et al. [52] for folate intake assessment, where a questionnaire including 81 questions produced a higher correlation than did a 140-question questionnaire.

The need for a carefully planned food product list is especially evident while considering the Japanese women population, as among the products that contribute to folate intake in these population are green tea, rice, young shoots of bracken, natto, miso, soy sauce, and purple laver, all foods that are consumed only rarely or not at all by European populations [55]. However, also in comparison with questionnaires applied for other European populations, there are products not consumed in countries of Central and Eastern Europe, such as Bovril and Marmite, which are included in questionnaire applied in Great Britain [57]. This case indicates the need to apply dedicated food frequency questionnaires for each population, such as Fol-IC-FFQ designed for a Polish population, and which may be also used for other countries of Central or Eastern Europe, after the necessary adjustments, if needed.

The possibility of using a questionnaire designed for another population is limited especially if there are different products contributing mainly to folate intake and responsible for variation of the folate intake in the population. In the study by Ishihara et al. [55] focusing on a group of Japanese women, depending on the assessed cohort, either green tea or spinach leaves were demonstrated to be the most important source of folate, while green tea contributed to the highest variation of its intake, and this was dependent on the individual preference for this product. At the same time, in the study by Bacardí-Gascón et al. [53] conducted in Mexico, among the most important predictors of folate intake were corn tortillas and papayas, rarely consumed by European populations, as in the case with green tea. As a result, green tea, corn tortillas and papayas were not included in Fol-IC-FFQ, but the spinach leaves were, as in Poland they are chosen more frequently than the other products indicated above.

Except for the length of the form and included food products, the observed validity and reproducibility are the most important issues. Considering the methods applied in the assessment of validity and reproducibility, it must be emphasized that the Bland-Altman method is the primary one [57]. When comparing obtained results with results of other studies, the majority of these did not include the Bland-Altman method, but only the kappa statistic [33,34], analysis of correlations [33,34,35,50,51,55,56,57], as well as assessment of individual distributions into tertiles [57] or quartiles [33,34,35].

In the own study, the analysis of the kappa statistic, analysis of correlations, as well as assessment of individual distributions into tertiles were also applied. Moreover, the share of individuals classified into the same and into the conflicting folate intake adequacy category were analysed. It was observed that for the analysis of the folate intake adequacy, the Fol-IC-FFQ may be a good method, both in the assessment of validity and reproducibility, as for over 70% of individuals, the results were valid in comparison with the reference method, and for almost 90% of individuals, they were reproducible. As a result, it must be indicated that the designed Fol-IC-FFQ may allow to indicate, in the population, individuals characterized by adequate folate intake and by inadequate folate intake.

The Bland-Altman method was applied by Galván-Portillo et al. [54] in the assessment of the reproducibility of a 127-item food frequency questionnaire to assess folate intake, as well as by Zekovic et al. [17] in the assessment of the validity of a 90-item food frequency questionnaire for folate (F-FFQ). In the study by Galván-Portillo et al. [48], the number of individuals observed to be within the LOA value was 45 out of 48, which confirmed a Bland-Altman index of 6.25%, higher than the value of five percent which is commonly assumed as a maximum value for the positive validation of a method of measurement [33]. Simultaneously, in the study by Zekovic et al. [17], a Bland-Altman index of 3.98% indicated a good agreement between methods in the comparison of the F-FFQ and three 24-h dietary recalls. The results observed in the own study in the assessment of the Fol-IC-FFQ are comparable with the results of Zekovic et al. [17] and Galván-Portillo et al. [54], but the reproducibility in own study was observed to be higher (Bland-Altman index of 2.7%), while the validity was stated to be borderline significant (Bland-Altman index of 5.3%). The results must be recognized as promising also for other countries of Central and Eastern Europe. Not only quite good validity is indicated, but also very good reproducibility.

The results were also confirmed using in the assessment of reproducibility other methods than the Bland-Altman plot. The share of individuals classified into the same tertile was almost 75%, while weighted κ statistic indicated substantial agreement (0.67) and the correlation was significant (*p* = 0.0000; *R* = 0.7995). At the same time, in the assessment of validity, the share of individuals classified into the same tertile was lower, at 40%, while weighted κ statistic indicated a slight agreement (0.19).

The reproducibility higher than validity, indicates that the obtained Fol-IC-FFQ may be a valuable method especially while the repeated measurements are conducted. It may be used, for example, during nutritional education, while the results of education must be verified, in order to indicate if the individuals sufficiently increased their folate intake. Similar conclusions were drawn for the Block DFE Screener to assess folate intake in the United States of America population assessed by Owens et al. [50], as it was demonstrated that the Block DFE Screener is especially useful in the repeated measurement of single nutrient intake.

However, not all studies of other authors have revealed such a good agreement for folate-specific food frequency questionnaires. For example, the food frequency questionnaire validated by Pufulete et al. [57] was stated to be a useful method for assessing folate intake, but mainly in male, not female respondents. Considering the fact that for female respondents, folate intake assessment is especially needed, questionnaires enabling reliable estimation of folate intake for women, such as Fol-IC-FFQ, are of great value. In the future, further analysis of the Fol-IC-FFQ may be needed, while food products that are consumed by a small number of respondents and that do not contribute high levels of folate may be excluded to simplify the questionnaire, as is also recommended by authors of other questionnaires [58].

The need for efforts to increase folate intake among all young women is also emphasized by other authors and dedicated food frequency questionnaires are considered valuable tools in the assessment of folate intake [53]. This is especially important, given that independently from the applied recommended level, folate intake in developed countries is insufficient in many young women.

## 5. Conclusions

Assessment of the Fol-IC-FFQ revealed a satisfactory level of validity and very good level of reproducibility in the population of young Polish women. The Fol-IC-FFQ may be considered a valid tool for the assessment of folate intake in young Polish women and a promising tool for the assessment of folate intake in young women in Central and Eastern Europe.

## Figures and Tables

**Table 1:** The scheme of an applied food frequency questionnaire including food products, serving sizes and frequencies in the Folate-Intake Calculation-Food Frequency Questionnaire (Fol-IC-FFQ).

Products	Serving Size	Frequency	Number of Servings *
Fish and fish products	50 g (deck of cards)	monthly	
Pasta, rice, groats	100 g of cooked (2/3 of a glass)	monthly	
Bean, soybeans, peas	100 g of cooked (2/3 of a glass)	monthly	
Nuts and seeds	15 g (1 spoon)	monthly	
Grains, wheat bran and germs	10 g (1 tablespoon)	monthly	
Milk, dairy beverages, cream	250 g (1 glass)	weekly	
Rennet cheese	20 g (thin slice)	weekly	
Cottage cheese, curd cheese, fromage frais, dairy desserts	40 g (1 slice, large tablespoon)	weekly	
Egg	50 g (1 egg)	weekly	
Egg yolk	30 g (1 egg yolk)	weekly	
Liver	100 g (palm of small hand)	weekly	
Other meat and offal	100 g (palm of small hand)	weekly	
Pate	40 g (1 tablespoon, 1 slice)	weekly	
Other cold cuts	15 g (thin slice of ham, 3 slices of sausage, 1/3 of wiener)	weekly	
Bread	35 g (1 medium slice, small roll)	weekly	
Oat, wheat, rye cereals, muesli	10 g (1 tablespoon)	weekly	
Fluor added to dishes	10 g (1 tablespoon)	weekly	
Corn flakes, corn crunches, puffed rice	10 g (2 tablespoons)	weekly	
Potatoes	70 g (1 medium, 3 tablespoons of puree)	weekly	
Broccoli, kale, Brussels sprouts, broad bean, asparagus, parsley, spinach	100 g (half of a glass, 1 glass of leafy vegetables)	weekly	
Zucchini, chicory, corn, red pepper, cauliflower, leek, green cabbage, parsnip, green peas, green beans, lettuce, beetroot	100 g (half of a glass, 1 glass of leafy vegetables)	weekly	
Celery, sorrel, cucumber, onion, eggplant, turnip, turnip cabbage, radish, pumpkin, carrot, tomato, red cabbage, green pepper	100 g (half of a glass, 1 glass of leafy vegetables)	weekly	
Avocado	70 g (half of medium one)	weekly	
Other fruits	100 g (half of a glass)	weekly	
Chocolate	20 g (3–4 chocolate bar squares)	weekly	

* Column completed by the respondents.

**Table 2:** The content of folate in one serving of a size specified in the Fol-IC-FFQ.

Products	Serving Size	Folate Content/Serving (µg)
Fish and fish products	50 g (deck of cards)	5
Pasta, rice, groats	100 g of cooked (2/3 of a glass)	12
Bean, soybeans, peas	100 g of cooked (2/3 of a glass)	69
Nuts and seeds	15 g (1 spoon)	9
Grains, wheat bran and germs	10 g (1 tablespoon)	21
Milk, dairy beverages, cream	250 g (1 glass)	11
Rennet cheese	20 g (thin slice)	5
Cottage cheese, curd cheese, fromage frais, dairy desserts	40 g (1 slice, large tablespoon)	8
Egg	50 g (1 egg)	32
Egg yolk	30 g (1 egg yolk)	30
Liver	100 g (palm of small hand)	317
Other meat and offal	100 g (palm of small hand)	10
Pate	40 g (1 tablespoon, 1 slice)	14
Other cold cuts	15 g (thin slice of ham, 3 slices of sausage, 1/3 of wiener)	1
Bread	35 g (1 medium slice, small roll)	12
Oat, wheat, rye cereals, muesli	10 g (1 tablespoon)	7
Fluor added to dishes	10 g (1 tablespoon)	5
Corn flakes, corn crunches, puffed rice	10 g (2 tablespoons)	1
Potatoes	70 g (1 medium, 3 tablespoons of puree)	14
Broccoli, kale, Brussels sprouts, broad bean, asparagus, parsley, spinach	100 g (half of a glass, 1 glass of leafy vegetables)	150
Zucchini, chicory, corn, red pepper, cauliflower, leek, green cabbage, parsnip, green peas, green beans, lettuce, beetroot	100 g (half of a glass, 1 glass of leafy vegetables)	64
Celery, sorrel, cucumber, onion, eggplant, turnip, turnip cabbage, radish, pumpkin, carrot, tomato, red cabbage, green pepper	100 g (half of a glass, 1 glass of leafy vegetables)	26
Avocado	70 g (half of medium one)	43
Other fruits	100 g (half of a glass)	15
Chocolate	20 g (3–4 chocolate bar squares)	2

**Table 3:** The folate intake calculated using 3-day dietary record and Fol-IC-FFQ, accompanied by share of individuals characterized by adequate or inadequate intake.

			3-Day Dietary Record	Fol-IC-FFQ1	Fol-IC-FFQ2
Mean (µg)			307	262	273
Standard deviation (µg)			119	132	136
Median (µg)			291 ^a,^*	226 ^b,^*	238 ^b,^*
Minimum (µg)			117	94	100
Maximum (µg)			845	903	869
Share of individuals characterized in comparison with recommendation by Jarosz [39]	adequate intake	*n*	27	15	19
		(%)	36.0	20.0	25.3
	inadequate intake	*n*	48	60	56
		(%)	64.0	80.0	74.7

* Distribution different than normal (verified using Shapiro-Wilk test, *p* ≤ 0.05); ^a,b^ values marked with different letters vary significantly on the basic of U Mann-Whitney test criteria pair-comparison; Fol-IC-FFQ1: food frequency questionnaire filled out directly after conducting 3-day dietary record; Fol-IC-FFQ2: food frequency questionnaire filled out six weeks after Fol-IC-FFQ1.

**Table 4:** The number and share of individuals classified into the same tertile and misclassified, as well as individuals of the same or conflicting folate intake adequacy category in comparison of Fol-IC-FFQ and 3-day dietary record.

		Fol-IC-FFQ1 vs. 3-Day Dietary Record	Fol-IC-FFQ1 vs. Fol-IC-FFQ2
Individuals classified into the same tertile	*n*	30	56
	%	40.0	74.7
Individuals misclassified (classified into opposite tertiles)	*n*	9	3
	%	12.0	4.0
Weighted κ statistic		0.19	0.67
Individuals of the same folate intake adequacy category	*n*	53	65
	%	70.7	86.7
Individuals of the conflicting folate intake adequacy category	*n*	22	10
	%	29.3	13.3

Fol-IC-FFQ1: food frequency questionnaire filled out directly after conducting 3-day dietary record; Fol-IC-FFQ2: food frequency questionnaire filled out six weeks after Fol-IC-FFQ1.
